# Evaluation of Human Platelet Lysate as an Alternative to Fetal Bovine Serum for Potential Clinical Applications of Stem Cells from Human Exfoliated Deciduous Teeth

**DOI:** 10.3390/cells13100847

**Published:** 2024-05-16

**Authors:** Ji-Young Yoon, Huong Thu Vu, Jun Hee Lee, Ji-Sun Shin, Hae-Won Kim, Hae-Hyoung Lee, Jong-Bin Kim, Jung-Hwan Lee

**Affiliations:** 1 Institute for Stem Cell & Matters, Cell & Matter Corporation, Cheonan 31116, Republic of Korea; wisdom7970@gmail.com (J.-Y.Y.); junheelee@dankook.ac.kr (J.H.L.); kimhw@dankook.ac.kr (H.-W.K.); 2 Institute of Tissue Regeneration Engineering (ITREN), Dankook University, 119 Dandae-ro, Cheonan 31116, Republic of Korea; huong.vuthudr@gmail.com (H.T.V.); haelee@dku.edu (H.-H.L.); 3 Department of Pediatric Dentistry, Faculty of Odonto-Stomatology, University of Medincine and Pharmacy at Ho Chi Minh City, Ho Chi Minh City 17000, Vietnam; 4 Department of Pediatric Dentistry, College of Dentistry, Dankook University, 119 Dandae-ro, Cheonan 31116, Republic of Korea; pedoshin@dankook.ac.kr; 5 Department of Biomaterials Science, College of Dentistry, Dankook University, 119 Dandae-ro, Cheonan 31116, Republic of Korea; 6 Department of Nanobiomedical Science & BK21 FOUR NBM Global Research Center for Regenerative Medicine, Dankook University, 119 Dandae-ro, Cheonan 31116, Republic of Korea; 7 UCL Eastman-Korea Dental Medicine Innovation Centre, Dankook University, 119 Dandae-ro, Cheonan 31116, Republic of Korea; 8 Mechanobiology Dental Medicine Research Center, Dankook University, 119 Dandae-ro, Cheonan 31116, Republic of Korea; 9 Cell & Matter Institute, Dankook University, 119 Dandae-ro, Cheonan 31116, Republic of Korea

**Keywords:** stem cells derived from human exfoliated deciduous teeth, human dental pulp stem cells, human platelet lysate, SHED, DPSC, hPL, cell therapy, FBS alternative, clinical application of stem cells

## Abstract

In recent years, there has been a surge in demand for and research focus on cell therapy, driven by the tissue-regenerative and disease-treating potentials of stem cells. Among the candidates, dental pulp stem cells (DPSCs) or human exfoliated deciduous teeth (SHED) have garnered significant attention due to their easy accessibility (non-invasive), multi-lineage differentiation capability (especially neurogenesis), and low immunogenicity. Utilizing these stem cells for clinical purposes requires careful culture techniques such as excluding animal-derived supplements. Human platelet lysate (hPL) has emerged as a safer alternative to fetal bovine serum (FBS) for cell culture. In our study, we assessed the impact of hPL as a growth factor supplement for culture medium, also conducting a characterization of SHED cultured in hPL-supplemented medium (hPL-SHED). The results showed that hPL has effects in enhancing cell proliferation and migration and increasing cell survivability in oxidative stress conditions induced by H_2_O_2_. The morphology of hPL-SHED exhibited reduced size and elongation, with a differentiation capacity comparable to or even exceeding that of SHED cultured in a medium supplemented with fetal bovine serum (FBS-SHED). Moreover, no evidence of chromosome abnormalities or tumor formation was detected. In conclusion, hPL-SHED emerges as a promising candidate for cell therapy, exhibiting considerable potential for clinical investigation.

## 1. Introduction

Cell therapy products are currently under active investigation for the treatment of diverse conditions, including cancer, leukemia, and various rare diseases, alongside tissue and organ regeneration [1,2,3]. Mesenchymal stem cells, such as adipose-derived stem cells (ADSCs), bone marrow-derived stem cells (BMSCs), and umbilical cord blood stem cells (UCBSC), have emerged as promising candidates owing to their capabilities of multilineage differentiation (osteogenesis, chondrogenesis, adipogenesis, etc.), self-renewal, and mild immunogenicity. With the advent of various stem cell candidates, including dental pulp stem cells (DPSCs) and stem cells from human exfoliated deciduous teeth (SHED), which originate from the neural crest [4,5], there is a growing interest in their applications, particularly due to their non-invasive procurement methods without pain and their demonstrated enhanced potential for neural differentiation, alongside possessing typical stem cell properties [6,7,8]. Of particular significance are SHEDs, derived from young donors, exhibiting superior proliferative capacity [9], along with remarkable differentiation potential encompassing angiogenesis [10] and osteogenesis [9], when compared to DPSCs. Additionally, SHEDs offer ample opportunities for tissue retrieval to isolate stem cells, whereas umbilical cord blood cells, despite being another sourced from young donors, become unattainable due to the limited acquisition window at birth [7]. Therefore, SHED holds significant promise for the future treatment of various diseases and tissue regeneration, positioning it as a leading candidate for cell therapy products.

In the field of cell therapy, stem cells need to be cultured and expanded when limited or significant quantities of tissue are required. FBS has emerged as the most prevalent growth factor supplement for this purpose. However, xenoantigens from FBS, serving as potential immunogens, may compromise therapeutic efficacy. This leads to persistent efforts to refrain from utilizing animal-derived materials in the production of cell therapy products, with a critical emphasis on verifying the absence of any residual FBS in these final products [11,12]. Research is ongoing to identify viable alternatives to FBS, with an emphasis on animal protein-free systems such as chemically defined media. However, there is still uncertainty about the most effective formula with relevant factors to ensure optimal cell adhesion and proliferation [13,14]. For this reason, formulating a universally applicable medium across diverse cell types remains a challenge.

Human platelet lysate (hPL) is a medium supplement approved by the US FDA, containing a higher concentration of growth factors (GFs) than other cell culture supplements, including platelet-rich plasma and fetal bovine serum (FBS) [15,16,17,18]. Numerous studies have reported that hPL is more effective than FBS in promoting cell expansion and enhancing differentiation across various cell types [19].

In this study, an analysis was undertaken to assess the effects of hPL on promoting cell proliferation and migration, as well as enhancing cell survivability in ROS conditions, aiming to explore hPL’s potential as an alternative to FBS. Furthermore, the differentiation ability and safety profiles of hPL-SHED were analyzed for clinical applications.

## 2. Materials and Methods

### 2.1. The Primary Culture of SHED

Exfoliated deciduous teeth were extracted from a six-year-old male to optimize the concentration of FBS and hPL and collected from five- and eight-year-old male subjects for subsequent experiments, following routine clinical extraction procedures at the Pediatric Department, Dental Hospital, Dankook University, under approved ethical guidelines (IRB number DKUDH 2021-12-002) sanctioned by the Institutional Review Board of Dankook University Dental Hospital. All participants provided written informed consent.

Following tooth extraction, stem cells from human exfoliated deciduous teeth (SHEDs) were isolated using previously described methods [20,21]. In brief, the dental pulp was digested in a solution of 2 mg/mL Collagenase type I (Worthington Biochemical, Lakewood, NJ, USA) and 4 mg/mL Dispase II (Invitrogen, Carlsbad, CA, USA) for 1 h at 37 °C in a water bath after mincing. The cell suspension was filtered using a 70 μm strainer after dilution with alpha MEM and then centrifuged. To optimize the concentrations of FBS and hPL for SHEDs, cells were suspended and cultured in a basal medium (αMEM (WelGENE, Daegu, Republic of Korea) supplemented with 2 mM L-glutamine (GlutaMAXTM-1 100X, Gibco, Thermo Fisher Scientific, Waltham, MA, USA), 0.1 mM L-ascorbic acid phosphate FUJIFILM Wako Pure Chemical Corporation, Osaka, Japan), 1% PS (Gibco, Thermo Fisher) containing 15% FBS (FBS, Corning, Corning, NY, USA) from passage 0 before seeding for experiments. For subsequent experiments, SHEDs separated from one donor were suspended and cultured separately in basal medium containing either 15% FBS or 5% human platelet lysate (hPL, STEMCELL Technologies, Vancouver, BC, Canada). The cells were incubated at 37 °C in an atmosphere containing 5% CO_2_ at 95% humidity. The medium was replaced every 2 days, and the cells were sub-cultured after 10 to 14 days, continuing in their respective growth media (contained with either 15% FBS or 5% hPL) before use. In addition, hPL-SHEDs were cultured in growth media supplemented with either 2% or 5% hPL for at least 1–2 passages before further experimentation. Given our focus on clinical applications, we abstained from utilizing flow cytometry for sorting.

### 2.2. The Optimization of FBS and hPL Concentration

SHEDs (passage 6–7) cultured in 15% FBS-containing growth medium from passage 0 were seeded into 96-well plate at a density of 5 × 10^2^ cells/well in basal medium supplemented with either FBS (15% and 10%) or hPL (2%, 5%, 7.5%, and 10%). At days 2, 4, and 6, the medium containing 10% CCK-8 (Dojindo, Kumamoto, Japan) solution in fresh serum-free αMEM was replaced in each well of the plate, followed by incubation for 2 h at 37 °C. Then, the absorbance of each well at 450 nm was recorded using a microplate reader (Thermo Fisher VarioskanTM LUX, Waltham, MA, USA). In each group, cell viability (%) was normalized with an average absorbance of 10% FBS-SHED on day 2. Cell survival was also examined by Live and Dead staining (0.5 µM calcein AM and 2 µM ethidium homodimer-1 solutions, Thermo Fisher, Waltham, MA, USA), and images were taken using an optical microscope (IX71, Olympus, Tokyo, Japan).

### 2.3. Cell Morphology

To evaluate the influence of hPL on cell size and shape, FBS-SHEDs were cultured in growth media supplemented with 10% FBS, and hPL-SHEDs were cultured in growth media supplemented with 2% or 5% hPL at a density of 4 × 10^4^ cells/well (6-well plate). After 24 h of incubation at 37 °C, cells were fixed with 4% paraformaldehyde (Tech & Innovation, Gangwon, Republic of Korea) at room temperature for 15 min and stained with Phalloidin (Alexa FluorTM 488, Thermo Fisher) and DAPI (4′,6-diamidino-2-phenylindole, Thermo Fisher). The stained images were taken using an optical microscope (IX71, Olympus, Tokyo, Japan). The cell roundness and area measured by ImageJ software 1.54f (Wayne Rasband, NIH, Bethesda, MD, USA) were used to compare the difference in cell morphology.

### 2.4. Proliferation and Self-Renewing Capacity

To compare the effect of FBS and hPL on the growth and replication of SHED, doubling time and colony-forming units (CFU) assays were conducted. In the doubling time assay, 5 × 10^4^ FBS-SHEDs plated on culture dishes (60 mm culture dish, SPL) with 3 mL of 10% FBS or hPL-SHEDs in 2% hPL or 5%hPL were incubated for 24, 48, and 72 h (1, 2, and 3 days). Cell doubling time was calculated based on the number of the counted cells on days 1, 2, and 3 and the following formula:

Doubling time = duration × ln (2)/ln (final number of cells/initial number of cells)

To perform the CFU assay, 500 cells were seeded in 5 mL of growth media (100 mm culture dish). After 10 days, cells were fixed with 4% paraformaldehyde (Merck, Darmstadt, Germany) for 15 min and then stained with 2.5 mg/mL crystal violet solution (Sigma-Aldrich, St. Louis, MO, USA) in 20% methanol for 30 min. After washing cells with PBS to remove excessive crystal violet solution, stained dishes were scanned to determine the staining intensity (Epson, Epson perfection v300 photo, Nagano, Japan), and the number of colonies was manually identified using a microscope.

### 2.5. Cell Migration Assay

To assess the impact of hPL on cell migration, 4 × 10^4^ FBS-SHEDs and hPL-SHEDs were seeded in the upper chamber of transwell inserts (Corning, NY, USA) with 8 μm pores using 100 μL of serum-free αMEM. The lower chamber (24-well plate, SPL) was filled with 350 μL of αMEM supplemented with either FBS or hPL. Specifically, the lower chamber for FBS-SHEDs was supplemented with either 10% FBS or 5% hPL, while the lower chamber for hPL-SHEDs was supplemented with 2%, 5%, or 10% hPL, or 10% FBS. Following incubation at 37 °C with 5% CO_2_ for 6 hours, the SHEDs were fixed using 4% paraformaldehyde (Merck) for 15 min and stained with a 2.5 mg/mL crystal violet solution (Sigma-Aldrich) in 20% methanol for 30 min. After washing with PBS 1X to remove excess crystal violet solution, the cells were stained with DAPI. Any SHEDs attached to the upper surface of the transwell were removed using cotton swabs, while those on the lower surface of the chamber (migrated cells) were visualized under an inverted microscope (IX71, Olympus, Tokyo, Japan) at ×100 magnification.

### 2.6. Cell Protective Effect of hPL under ROS Condition Induced by Hydrogen Peroxide (H_2_O_2_)

The cell-protective effect of hPL under ROS conditions induced by hydrogen peroxide (H_2_O_2_) was investigated. To assess the protective effect of different culture conditions, 10% FBS-SHEDs, as well as 5% and 2% hPL-SHEDs, were seeded at a density of 5 × 10^3^ cells/well in 96-well plates (SPL) and cultured separately in the same medium as the pre-cultured medium. Following overnight incubation at 37 °C, cells were exposed to varying concentrations of H_2_O_2_ (100, 200, 400 µM). After 24 h, 100 µL of CCK-8 solution was added and incubated for 2 h at 37 °C. The absorbance of the resulting orange product was measured at 450 nm using a microplate reader. The percentage of cell viability in each group was calculated relative to their respective controls (H_2_O_2_-untreated groups). Live and Dead cell assessments were also conducted in all groups. The culture medium was incubated with 2 μM calcein-AM and 4 μM PI (Invitrogen, Waltham, MA, USA) for 20 min, followed by observation under a fluorescent microscope (IX71, Olympus, Tokyo, Japan). Live and Dead cells were distinguished by the conversion of non-fluorescent calcein-AM to a fluorescent dye by intracellular esterase enzymes in living cells and the uptake of propidium iodide (PI) dye by dead cells. Cells without H_2_O_2_ treatment served as negative controls.

To determine whether the protective ability against cell death originates from the cells or the culture medium, the supplements of the media were switched, and the same experiments were conducted as described above.

### 2.7. Analysis of Chromosomal Microarray-Cytogenetic Microarray (CMA)

A cytogenetic microarray (CMA) including chromosome numbers and G-banded and C-banded karyotypes of SHEDs (Passage 10) cultured both in 5% hPL and 10% contained medium was performed by Dx&Vx (Seoul, Republic of Korea).

### 2.8. Flow Cytometric Analysis

To ascertain the surface markers of hPL-SHEDs and FBS-SHEDs, flow cytometry analysis was conducted employing specific markers including CD14, CD29, CD44, and CD73 (obtained from Thermofisher, catalog numbers 11-0149-42, 11-0299-42, 11-0441-82, and 11-0739-42, respectively). Analysis was performed using a FACS Caliber instrument (Becton-Dickinson, Milpitas, CA, USA).

### 2.9. SHED Differentiation Assays

Quantities of 10% FBS-SHEDs and 2% and 5% hPL-SHEDs were seeded and cultured separately overnight under their respective conditions. The following day, SHEDs were exposed to the same differentiation conditions using commercial kits.

To initiate osteogenic differentiation, a density of 1 × 10^4^ cells was seeded onto a 24-well plate and allowed to adhere overnight. Osteogenic differentiation was then induced by replacing the culture medium with StemPro^®^ Osteogenic Differentiation Media (Gibco, Thermo Fisher) and maintained until day 14. On day 7, following cell fixation, alkaline phosphatase (ALP) staining was conducted using FAST BCIP/NBT (B5655, Sigma) for 1 h at 37 °C. On day 14, a 40 mM solution of Alizarin Red S (A5533, Sigma Aldrich) at pH 4.2 was applied to the fixed cells for 30 min to confirm mineralization.

Adipogenic differentiation was induced by the incubation of SHED in StemPro^®^ Adipogenesis differentiation media (Gibco, Thermo Fisher). Cells were seeded at a density of 10,000 cells/well (24-well plate, SPL) and then changed to differentiation media when the cell reached 90% confluence. Differentiation media were changed every 3 days until day 14. Cells’ accumulated intracellular lipids were analyzed using Oil Red O staining. After 14 days of differentiation, the cells were washed with PBS and fixed in 4% PFA for 30 min at room temperature. The PFA was removed, and the cells were rinsed with PBS. Then, 60% isopropanol (Nakalai Tesque, Kyoto, Japan) was added and incubated for 5 min at room temperature. After aspirating isopropanol, the cells were stained with Oil Red O solution (0.5% Oil Red O in isopropanol, Sigma-Aldrich), and the stock solution was further diluted 3:2 in distilled water for 15 min. After staining, the cells were washed with distilled H_2_O_2_ at least three times, and images were captured. For staining quantification, Oil Red O stain was extracted with 100% isopropanol for 5 min with gentle rocking. The absorbance of extracted Oil Red O stain was measured at 492 nm using a microplate reader.

For chondrogenesis, SHEDs were plated at a density of 5 × 10^4^/10 μL on 24-well plate and incubated for 6 h at 37 °C to allow cell attachment. After 6 h, 500 uL growth media (10% FBS, 2% and 5% hPL) was added. The next day, culture media was shifted to chondrogenic media (DMEM, Dulbecco’s Modified Eagle’s Medium, high glucose, Welgene) containing 1% Insulin-Transferrin-Selenium (Gibco, Thermo Fisher), 1% PS, 10 mM transforming growth factor-β1 (TGF-β1, Peprotech, Cranbury, NJ, USA), and 50 μg/mL Ascorbic acid for 28 days at 37 °C in 5% CO_2_. The media was changed every 3 days, and Alcian blue staining (Sigma-Aldrich) was performed on day 28.

Neurogenic differentiation was conducted by seeding 2 × 10^3^ cells/well (24-well plate) in 500 μL growth media, followed by changing into neuronal induction media (NeurobasalTM Plus medium supplemented with 2% B27 (Gibco, Thermo Fisher), 1% P/S, 1% L-GlutaMAX, 20 ng/mL bFGF (recombinant human basic fibroblast growth factor, Peprotech), and 20 ng/mL hEGF (Animal-Free Recombinant Human EGF, Peprotech)) after 16–24 h. The induction media was changed partially every 3 days.

### 2.10. Immunocytochemistry (ICC)

To detect the expression of specific proteins related to neurogenic differentiation, immunocytochemistry (ICC) was performed. After 7 days culturing in neurogenic differentiation medium, cells were fixed with 4% PFA and incubated with 2% Bovine Serum Albumin (BSA, Solmate^TM^, Seoul, Republic of Korea) for 30 min to suppress nonspecific staining, followed by cell permeabilization with 0.1% Triton^TM^ X-100 (Sigma-Aldrich). Thereafter, cells were incubated with primary antibodies, GFAP (Glial fibrillary acidic protein, Agilent, Santa Clara, CA, USA), which constitutes a portion of the cytoskeleton in astrocytes, and Tubulin β 3 (TUBB3, purified anti-Tubulin β 3 Antibody, Biolegend, San Diego, CA, USA), which is the main component of microtubules, at a dilution of 1:400 overnight at 4 °C. Then, the samples were subsequently incubated with the second antibodies at a dilution of 1:300 overnight at 4 °C. The nuclei of the cells were counterstained with DAPI for 5 minutes. The cells cultured in growth media before changing to neurogenic media were used as control groups. The images of stained cells were taken by the inverted fluorescence microscope. The fluorescence intensity of stained cells was measured by ImageJ software 1.54f.

### 2.11. Quantitative Real-Time Polymerase Chain Reaction (qRT-PCR)

To evaluate the differentiation potential of hPL-SHEDs to odontoblasts/osteocytes, chondrocytes, adipocytes, and neuron-like cells, quantitative real-time polymerase chain reaction (qRT-PCR) was performed on days 3 and 7 of differentiation induction. The expression levels of genes related to odontoblastic/osteogenic differentiation (like alkaline phosphatase (*ALP*), dentin sialophosphoprotein (*DSPP*), dentin matrix protein 1 (*DMP1*), osteocalcin (*OCN*), and runt-related transcription factor 2 (*RUNX2*)), adipogenic differentiation (including peroxisome proliferators-activated receptor-γ2 (*PPAR-γ2*), fatty acid binding protein 4 (*FABP4*), and lipoprotein lipase (*LPL*)), chondrogenic differentiation (such as collagen type II A (*ColIIA*), *SOX9*, and Aggrecan (*ACAN*)), and neurogenic differentiation (like mammalian achaete-scute homolog-1(*MASH-1*), *neuroD1*, *Nestin*, glial fibrillary acidic protein (*GFAP*), and *β-tubulin*) were measured. The qRT-PCR primers and their sequences are listed in Table S1.

After cells were collected, mRNA was isolated using a Ribospin kit (GeneAll, Seoul, Republic of Korea) according to the manufacturer’s instructions. For qRT-PCR, SensiMix™ SYBR Hi-ROX kit (Bioline, QT605-05) with additional MgCl_2_ (BIO-37026, Bioline, London, UK) was used, and qRT-PCR was performed by StepOne™ Plus (Applied Biosystems, Waltham, MA, USA). The fold change of the gene expression was calculated by the comparative CT method (2^−ΔΔCT^) and normalized to an endogenous housekeeping gene, *GAPDH*. The results are expressed as mRNA relative expression.

### 2.12. Organ Toxicity and Tumor Formation Test In Vivo

The animal care and experimental protocols were approved by the Animal Care and Use Committee at Dankook University, Republic of Korea (#21-033). Nude mice (8 weeks old, male) were housed in a cage under controlled environmental conditions with ad libitum access to water and food. SHEDs were prepared at a density of 2 × 10^6^ cells/100 μL in PBS and administered subcutaneously using a 1 mL syringe under isoflurane inhalation anesthesia. Tumor formation at the injection site was monitored in the mice for 6 months. After 6 months of injection, organs, including the lung, spleen, kidney, heart, liver, brain, and skin, were harvested from each mouse, fixed in 10% neutral buffered formalin at room temperature, embedded in a paraffin block, sectioned, and stained with hematoxylin and eosin (H–E). The images were taken by a slide scanner (VS200, Olympus, Japan).

### 2.13. Statistical Analyses

Data were expressed as the mean ± standard deviation, and statistical analyses were performed using GraphPad Prism 8 software (San Diego, CA, USA). Statistical significance between groups was evaluated using one-way analysis of variance (ANOVA) or two-way ANOVA followed by Tukey’s multiple comparisons tests. *p* < 0.05 was considered significant.

## 3. Results

### 3.1. The Evaluation hPL as an Alternative to FBS in SHED Culture

#### 3.1.1. Optimization of hPL and FBS Concentration for SHED Culture

SHEDs were isolated from the incisor of a six-year-old male and cultured up to passage 5. Subsequently, SHEDs were cultured with either 10% or 15% FBS or varying concentrations (2%, 5%, 7.5%, and 10%) of hPL-containing medium for 6 days (see Figure S1A). Cell viability was assessed on days 2, 4, and 6 using Live and Dead and CCK assays (Figure S1B,C). The results showed that the cell viability of SHEDs increased with increasing concentrations of both FBS and hPL. hPL groups exhibited a higher proliferation capacity than FBS groups, even at lower concentrations, suggesting that hPL has a more significant effect on the proliferation of SHEDs. However, SHEDs cultured in 10% hPL showed lower viability levels on days 4 and 6 compared to those in lower concentrations of hPL. This difference may be attributed to cell detachment and death caused by excessive proliferation in a confined area. Given the highest and similar proliferation results observed between the 5% and 7.5% hPL groups, we chose the 5% hPL and compared it with 10% FBS in subsequent studies.

#### 3.1.2. The Effect of Cell Proliferation of hPL and FBS

**Figure 1:**
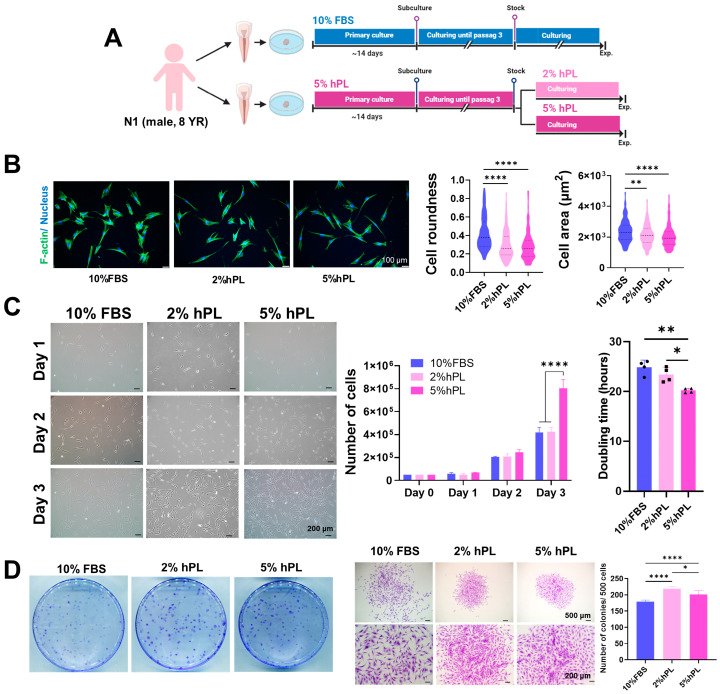
Comparison of cell morphology and proliferation effects between 10% FBS, 2% hPL, and 5% hPL. (**A**) Schematic representation of the experimental setup. Pulps isolated from two teeth obtained from one donor were cultured in media containing either 10% FBS or 5% hPL from the initial time point. Upon reaching passage 3, cells were frozen and subsequently cultured in media supplemented with 10% FBS, 2%, or 5% hPL for experimental purposes. (**B**) Cell morphologies in each group. F-actin and nucleus were stained with Phalloidin (green) and DAPI (blue), respectively. Cell roundness and area were analyzed using ImageJ software 1.54f with stained images. A total of 160 cells were analyzed, revealing a more spindle-shaped elongated morphology in the hPL groups, with no significant difference observed between the 2% and 5% hPL groups. (**C**) Proliferation effect. A total of 50,000 cells were seeded onto 60 mm culture plates. The number of cells was counted on days 1, 2, and 3, and the doubling time was calculated. The doubling time was found to decrease in the hPL groups, with shorter times observed at higher concentrations of hPL. (**D**) CFU (colony-forming units) assay for analysis of self-renewal capacity. A total of 500 cells were seeded onto 100 mm culture dishes, and the assay was conducted on day 10. Data are shown as mean ± SD (n = 3). * *p*  <  0.05, ** *p*  <  0.01, **** *p*  <  0.0001 using one-way analysis of variance (ANOVA) or two-way ANOVA followed by Tukey’s multiple comparison test.

We extracted two teeth from one single donor (N1, 8 year, male) and isolated SHEDs and cultured them in a medium containing 10% FBS and 5% hPL separately from the initial time. Subsequently, the SHED group was divided into 2% and 5% groups from passage 3 after the freezing–thawing step for further studies (Figure 1A). Cell morphology was analyzed using immunocytochemistry (ICC). hPL-SHEDs were observed to exhibit a smaller size and increased elongation compared to FBS-SHEDs (Figure 1B), in line with findings from previous studies [19]. Cell counts were performed daily for three days to assess doubling time. Results showed comparable cell numbers between the 10% FBS and 2% hPL groups, with the 5% hPL group exhibiting higher cell numbers, reaching nearly twice the count at day 3 (Figure 1C). The colony-forming unit (CFU) assay revealed that hPL-SHED demonstrated elevated colony counts, indicating an enhanced self-renewal capacity even at lower concentrations than FBS (Figure 1D). Remarkably, a marginally decreased colony count was noted at a 5% hPL concentration compared to 2% hPL, yet a slight increase in colony size was observed.

#### 3.1.3. The Effect of Cell Migration of hPL and FBS

**Figure 2:**
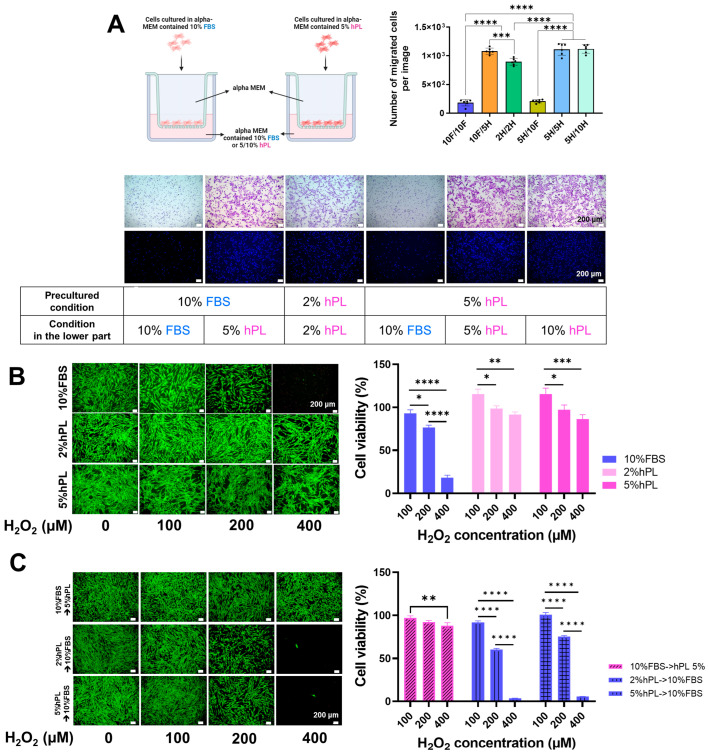
Effects of hPL and FBS on cell migration, and cell survival under oxidative stress. (**A**) Migration assay utilizing a transwell system. Quantities of 10% FBS-SHEDs or 5% Hpl-SHEDs were seeded with serum-free medium in the upper chamber, and medium containing 10% FBS or 5/10% hPL was added to the lower chamber. Both FBS-SHEDs and hPL-SHEDs exhibited migration toward the hPL-containing medium, while only minimal migration occurred toward FBS-containing media, indicating the cell recruitment capacity of hPL. (**B**) Protection against H_2_O_2_-induced reduction in cell viability. Cells were treated with H_2_O_2_ at concentrations of 0, 100, 200, and 400 μM for 24 h, and cell viability was assessed using Live and Dead staining (Green: Live cells; Red: Dead cells) and the CCK-8 assay. Cell viability decreased in FBS-supplemented medium in a concentration-dependent manner, significantly decreasing under 400 μM H_2_O_2_. Conversely, viability increased at 100 mM compared to the control (non-treated group) in both 2% and 5% hPL groups, reaching over 80–90% even at 400 mM. (**C**) Assessment of antioxidant ability following supplementation change from FBS to hPL or vice versa in the final culture condition, indicating that the antioxidative capability originates from hPL and is not retained under different culture conditions. Data are shown as mean ± SD (n = 3). * *p* < 0.05, ** *p* < 0.01, *** *p* < 0.001, **** *p* < 0.0001 using one-way analysis of variance (ANOVA) or two-way ANOVA followed by Tukey’s multiple comparison test.

Next, we assessed the migratory capacity of cells in various conditions. SHEDs cultured under different conditions were seeded with serum-free medium in the upper chamber of a transwell system, while media containing various concentrations of FBS or hPL were added to the lower chamber. After 6 h, the migrated cells were stained and quantified. As illustrated in Figure 2A, SHEDs demonstrated significantly enhanced migration toward the hPL-containing medium, irrespective of the preconditioned medium.

#### 3.1.4. The Cell Protection Effect of hPL and FBS against Oxidative Stress on Cells

To test the hypothesis that hPL was superior to FBS in preventing cell death under oxidative stress, SHEDs were exposed to different levels of H_2_O_2_ (100, 200, and 400 μM) in the presence of either FBS or hPL. The results revealed that the viability of FBS-SHED gradually decreased in correlation with the concentration of H_2_O_2_ and abruptly dropped when exposed to 400 μM H_2_O_2_. In contrast, hPL-SHED maintained viability of over 8–90% even when exposed to 400 μM H_2_O_2_ (Figure 2B). In addition, the protective effect against cell death induced by H_2_O_2_ changed depending on the final culture conditions when the medium was switched between hPL-SHED and FBS-hPL and subsequently exposed to H_2_O_2_ (Figure 2C).

### 3.2. Characterization of hPL-SHEDs and FBS-SHEDs

#### 3.2.1. Surface Markers and Chromosomal Stability

**Figure 3:**
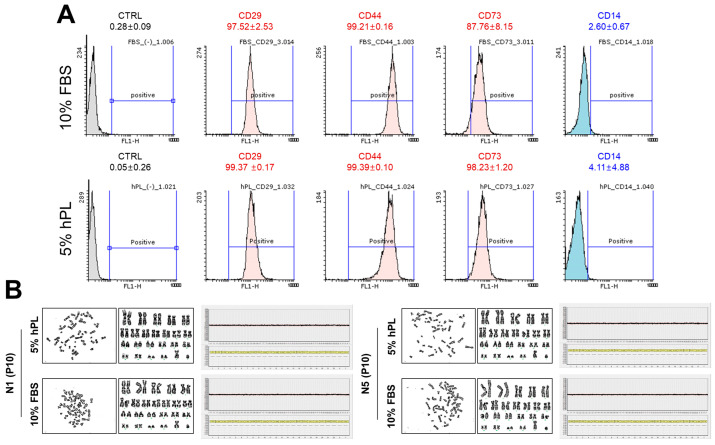
Characteristics of hPL-SHEDs and FBS-SHEDs. (**A**) Analysis of stem cell markers (CD29, 44, and 73 for positive markers and CD14 for negative makers) by flow cytometry. (**B**) Chromosomal stability was assessed by karyotyping and chromosomal microarray (CMA). Any abnormalities in the number or structure of chromosomes were not detected in passage 10 samples.

Flow cytometry analyses were carried out. The results demonstrated that 10% FBS-SHEDs and 5% hPL-SHEDs expressed MSC surface markers CD 29, 44, and 73 positively and rarely expressed the hematopoietic stem cell marker CD 14 [22,23], which proved that the majority of these cells were SHEDs (Figure 3A). To minimize donor-dependent effects, SHEDs were extracted and cultured from another donor (N5, 5 year, male) as well using the same methodology. Both FBS-SHEDs and hPL-SHEDs from the two donors exhibited no chromosomal abnormalities at passage 10 in Comparative Genomic Hybridization Microarray (CMA) and karyotype analysis. Furthermore, the analysis of DNA copy numbers using 1440 Whole Genome BAC clones from all groups showed normal patterns (Figure 3B).

#### 3.2.2. Differentiation Capacity of hPL-SHEDs and FBS-SHEDs

The differentiation capacity of stem cells was evaluated across various lineages, including osteogenic/odontogenic, chondrogenic, and adipogenic. To evaluate the impact of the culture conditions before differentiation and to mitigate the influence of supplements (FBS or hPL) during the differentiation process, differentiation media were selected using commercial kits that did not contain any common supplement such as FBS.

**Figure 4:**
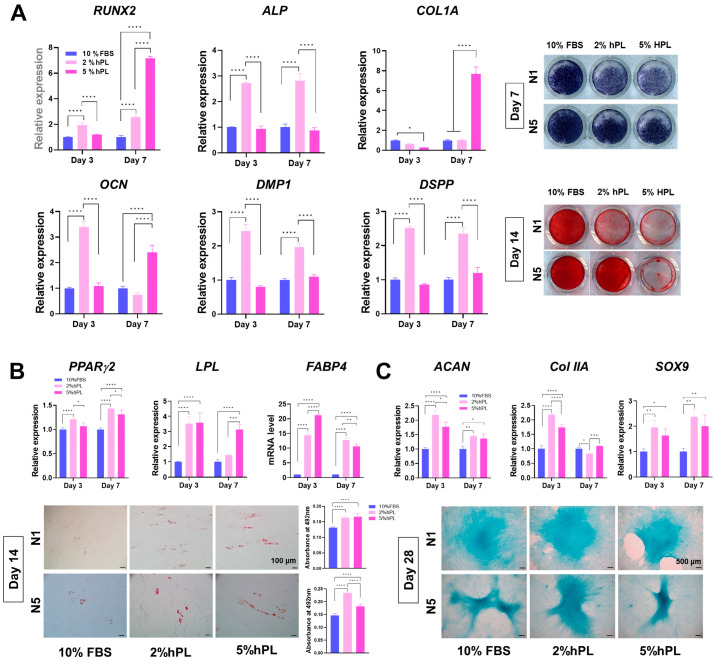

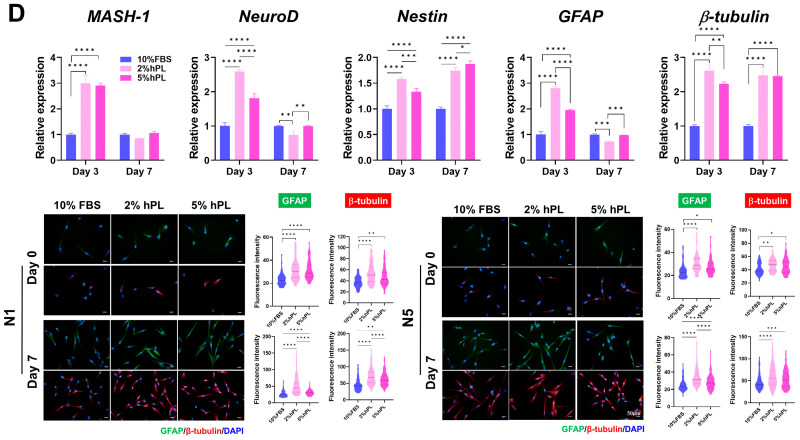
Cell differentiation capacity of 2/5% hPL-SHEDs and 10% FBS-SHEDs. All differentiation media were used as commercial products not containing FBS or hPL for the same differentiation condition. (**A**) Osteogenic (*RUNX2*, *ALP*, *ColA1*, *OCN*, *DMP1* and *DSPP*), (**B**) adipogenic (*PPARg2*, *LPL*, and *FABP4*), (**C**) chondrogenic (*ACAN*, *Col II A*, and *SOX9*), and (**D**) neurogenic (*MASH-1*, *Neuron D1*, *Nestin*, *GFAP*, and *b-tubulin*) gene expression levels were measured using qRT-PCR on days 3 and 7. (**A**) ALP and ARS Red S assays were performed to access ALP activity and mineralization on days 7 and 14, respectively. For the detection of neutral triglycerides, lipids, and polysaccharides, (**B**) Oil Red O and (**C**) Alcian blue staining were carried out on days 14 and 28, respectively. hPL-SHEDs exhibited significantly higher levels than FBS-SHEDs in most genes, although predominance varied between the 2% and 5% hPL groups. (**D**) The protein levels of GFAP and β-tubulin were assessed by ICC, revealing significantly higher expressions in the 2% hPL group. Data are shown as mean ± SD (n = 3). * *p* < 0.05, ** *p* < 0.01, *** *p* < 0.001, **** *p* < 0.0001 using one-way analysis of variance (ANOVA) or two-way ANOVA followed by Tukey’s multiple comparison test.

Initially, osteogenic/odontogenic differentiation was induced and assessed using qRT-PCR, ALP, and Alizarin Red S (ARS) staining. The results revealed that odontogenic genes including *RUNX2*, *ALP*, *Col1a*, *OCN*, *DMP1*, and *DSPP* were most highly expressed in the 2% hPL-SHEDs on both day 3 and day 7, except for *Col1A*, which exhibited the highest expression in the 10% FBS group on day 3 and in the 5% hPL group on day 7. The results of ALP and ARS staining, which were analyzed for ALP activity and mineralization, respectively, showed that 10% FBS-SHEDs exhibited the most intense staining, followed by 2% and 5% hPL-SHEDs (Figure 4A). For the evaluation of adipogenesis, the expression of related genes including PPAR-γ1, LPL, and FABP4 was investigated by q-RT PCR, and Oil Red O staining was performed. The results showed that 2% hPL-SHED expressed the highest level of genes and appeared in lipid droplets the most (Figure 4B). Under chondrogenic conditions, *ACAN*, *Col IIA*, and the *SOX9* genes were the most prominently expressed in the 2% hPL-SHEDs on both day 3 and day 7, while the levels of proteoglycan stained by Alcian blue (Figure 4C) were similar among groups. Neurogenic genes such as *MASH-1*, *NeuroD*, *Nestin*, *GFAP*, and *β-tubulin* were expressed higher in hPL-SHED groups, and the superiority between 2% and 5% hPL varied. Lastly, GFAP (glial fibrillary acidic protein) and β-tubulin (neuron-specific marker) levels were demonstrated in protein levels on day 7, and 2% HLP-SHEDs showed the highest level in both donor-derived SHEDs. In conclusion, the differentiation capacity of SHED-hPL is comparable to or significantly higher than that of FBS-SHED, particularly in neurogenic differentiation.

#### 3.2.3. In Vivo Studies of Tumorigenesis and Organ Toxicity

**Figure 5:**
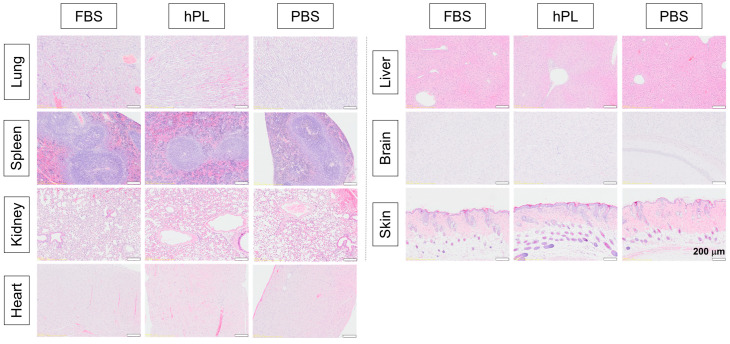
In vivo evaluation of organ toxicity and tumor formation. Representative histological images illustrating the absence of tumor formation and structural alterations in organs (lung, spleen, kidney, heart, liver, brain, and skin) of nude mice transplanted with 10% FBS-SHEDs, 5% hPL-SHEDs, or PBS for 6 months. No evidence of tumor formation was detected, and organs exhibited normal histology without observable injuries. (n = 5).

SHEDs were injected into the subcutaneous tissue of nude mice, and tumor growth was monitored for 6 months. As expected, no tumor-like tissues were detected in any organs, including the lung, spleen, kidney, heart, liver, brain, skin, and injection site of the cells (Figure 5).

## 4. Discussion

For the safe clinical application of stem cells, the exclusion of animal-derived components is imperative due to safety concerns [15,24,25]. Despite efforts to develop chemically defined serum-free media as an alternative to fetal bovine serum (FBS), challenges persist in creating media suitable for various cell types. Consequently, the use of human platelet lysate (hPL) has emerged as a promising alternative [26,27,28,29,30]. Although several comparative studies have been conducted, comprehensive investigations of cells cultured under each condition are scarce. This study assesses hPL as an alternative to FBS by investigating its impact on cell proliferation and migration, as well as examining its effects on surface markers and chromosome stability during high passaging. Furthermore, in anticipation of the effects following clinical application, the characteristics of cells cultured with hPL, including differentiation abilities and tumor formation potential, were investigated.

Considering the necessity of cell amplification for therapeutic purposes, chromosome stability at high passages is crucial [31,32]. Chromosomal Microarray Analysis (CMA) confirmed chromosomal integrity up to passage 10. Surface markers indicative of stem cell characteristics were positive, while hematopoietic stem cell markers were negative, confirming the stem cell phenotype in most cultured cells in both FBS-SHEDs and hPL-SHEDs.

In the proliferation test, the results demonstrated that cell numbers were doubled compared to both the 10% FBS and 2% hPL groups within 3 days under the 5% hPL condition, suggesting that 5% hPL provides the most favorable environment for proliferation. This finding holds significance for clinical application as it presents a cost-effective option owing to its heightened efficiency even at lower concentrations compared to fetal bovine serum (FBS). Moreover, colony formation significantly increased in both the 2% and 5% hPL groups compared to FBS-SHEDs. Interestingly, the 2% hPL group exhibited a greater number of colonies than the 5% hPL group, potentially attributed to relatively larger colony sizes observed at 5%, possibly due to the merging of colonies.

Migration assays demonstrated increased migration into hPL-supplemented media compared to FBS, regardless of prior culture medium supplementation, suggesting a higher concentration of growth factors in hPL influencing migration. Additionally, the absence of a difference between 5% and 10% hPL media may be attributed to limitations in the available surface area for cell attachment within the set timeframe. This indicates that hPL has a capacity for cell recruitment, while culturing condition (FBS or hPL) may not affect migration capacity in clinical conditions.

Various cell types produce reactive oxygen species (ROS), a group of oxygen-derived molecules including hydrogen peroxide (H_2_O_2_), free radical superoxide anion (O^2−^), and hydroxyl radical (OH^−^). ROS play a pivotal role in maintaining tissue homeostasis by regulating fundamental cellular activities such as cell death, differentiation, and proliferation. However, excessive oxidative stress can disrupt tissue function in various diseases by triggering cell apoptosis [33,34,35,36,37]. We evaluated cell viability under reactive oxygen species (ROS) conditions induced by H_2_O_2_ to assess the cell-protective effect of hPL during culture. The results showed that cell viability is higher in culture conditions containing hPL when exposed to H_2_O_2_ compared to those containing FBS. SHED exhibited nearly complete viability under high concentration of H_2_O_2_ (400 μM) in hPL-supplemented media, contrasting with significant cell death observed in FBS-supplemented media at high H_2_O_2_ concentrations. This observation suggests that the use of hPL can effectively shield SHEDs from the adverse effects of reactive oxygen species (ROS) during culture, as previously reported [38].

hPL, similar to FBS, constitutes a complex mixture of proteins, including serum albumin, hormones, vitamins, trace elements, and growth factors essential for cell attachment, proliferation, and maintenance. Typically, the total protein content of HPL and FBS exceeds 50–55 mg/mL and 38 g/L (ranging from 32 to 70 mg/mL), respectively, although batch-to-batch variations occur. Notably, HPL is reported to contain higher concentrations of growth factors, such as PDGF-AB, basic fibroblast growth factor (bFGF), transforming growth factor beta (TGF-ß1), insulin-like growth factor 1 (IGF-1), and vascular endothelial growth factor (VEGF), compared to FBS. In addition, human platelet lysate (hPL) contained antioxidative biomolecules, including superoxide dismutase, glutathione peroxidase, and catalase [19,38,39]. These may explain why SHEDs showed a higher cell proliferation rate, migration, and antioxidative effect under hPL-containing medium than the FBS one.

Next FBS-SHEDs and hPL-SHEDs were examined for osteo-/odonto-, adipo-, chondro-, and neurogenic differentiation abilities. Most of the osteo-/odontogenic genes revealed the highest levels in 2% hPL, while ALP activity and mineralization confirmed by ALP staining and ARS staining decreased in hPL-SHED groups compared to FBS-SHEDs. This suggests that the heightened proliferation potential induced by hPL in the culture medium before differentiation may hinder the osteo-/odontogenic differentiation process.

In adipogenesis, the hPL groups exhibited higher levels of adipogenic genes compared to FBS-SHEDs, along with increased neutral lipids stained with Oil Red O indicating differentiation into adipocytes.

The results of chondrogenic differentiation analyses showed higher expression levels of related genes in the hPL groups, while Alcian blue staining, visualizing chondrogenesis, was similar, despite the pellet being slightly denser in 5% hPL-SHEDs.

Finally, we investigated neurogenesis. DPSC/SHEDs are neural crest-derived stem cells [40,41,42,43]. For this reason, DPSC/SHEDs are well-known to have a high capacity for neurogenesis and neurotrophic effects [6,44,45]. Most neurogenic genes showed significantly higher levels in hPL-SHED groups than FBS-SHEDs. Consistent with these findings, GFAP and β-tubulin intensities similarly increased. Particularly, 2% hPL-SHEDs demonstrated the highest level in both gene and protein expression.

Based on the differentiation results, it is evident that hPL can positively influence the differentiation capacity of SHEDs in adipogenesis and neurogenesis, while showing comparability in chondrogenesis and less impact in odonto-/osteogenesis. Despite our investigation involving SHEDs from two donors, it is conceivable that these outcomes may be influenced by donor variability or the condition of the pulp. Therefore, extensive studies involving numerous cases are warranted to standardize the capacity of SHEDs cultured on hPL. Following thorough evaluation, SHEDs can be applied as a cell therapy according to the target disease.

In addition, growth factors (GFs) are not directly present in the liquid lysate but are encapsulated within exosomes and vesicles in HPL and FBS. Alongside GFs, exosomes and vesicles also contain miRNA, which plays a significant role in cellular communication [46]. In addition to the impact of differing growth factor concentrations, conducting exosomal small RNA-seq can offer additional insights into the composition of exosomes or small vesicles. This approach holds promise for advancing our understanding of the underlying mechanisms of this process.

In the realm of clinical application, it is imperative to ascertain the potential for tumorigenesis due to the pronounced proliferative and self-renewal capacities of stem cells. While mesenchymal stem cells containing DPSC/SHEDs generally entail a lower tumorigenic risk relative to pluripotent stem cells, the comprehensive confirmation of this distinction is requisite prior to clinical implementation [47]. Hence, 5% hPL-SHEDs were injected subcutaneously into nude mice and the injected site was observed for 6 months; no tumor formation was observed. Furthermore, histological analysis conducted to assess tissue toxicity after 6 months revealed no structural changes or injuries in any tissue, confirming the stability of hPL-SHEDs as a viable option for cell therapy in clinical applications.

In summary, hPL, as an alternative for FBS, demonstrated superior proliferation and migration abilities compared to FBS when incorporated into the SHED culture medium. hPL-SHEDs maintained stem cell characteristics effectively and did not exhibit abnormal chromosomes attributed to passaging or tumor formation in the in vivo study. While there were slight variations in differentiation ability, hPL is anticipated to be a promising candidate for cell therapy applications, especially when the target disease is carefully selected.

## 5. Conclusions

The medium supplemented with hPL markedly augmented both cell proliferation and migration, while also demonstrating a protective effect against oxidative stress. hPL-SHEDs have remarkable differentiation capabilities including adipo-, chondro-, and neurogenesis, especially in 2% hPL when compared to FBS-SHEDs, even though osteogenic differentiation was slightly lower. Moreover, hPL-SHEDs did not demonstrate any evidence of tumor formation or organ toxicity through an in vivo study, highlighting the safety and efficacy of this culture approach for potential clinical applications.

## Data Availability

The original contributions presented in the study are included in the article/Supplementary Material, further inquiries can be directed to the corresponding author/s.

## Supplementary Materials

The following supporting information can be downloaded at https://www.mdpi.com/article/10.3390/cells13100847/s1. Figure S1: Cell proliferation effect on SHEDs according to the concentration of hPL; Table S1: Primer sequences used in the qRT-PCR.
