# Supplementary material for: Evaluation of Human Platelet Lysate as an Alternative to Fetal Bovine Serum for Potential Clinical Applications of Stem Cells from Human Exfoliated Deciduous Teeth
Source: Cells. 2024 May 16;13(10):847. doi: 10.3390/cells13100847 (PMC11120611; doi:10.3390/cells13100847)
Supplement: Supplementary file 1 [file cells-13-00847-s001.zip › cells-2968431-supplementary.pdf]

Supplementary Figure and table

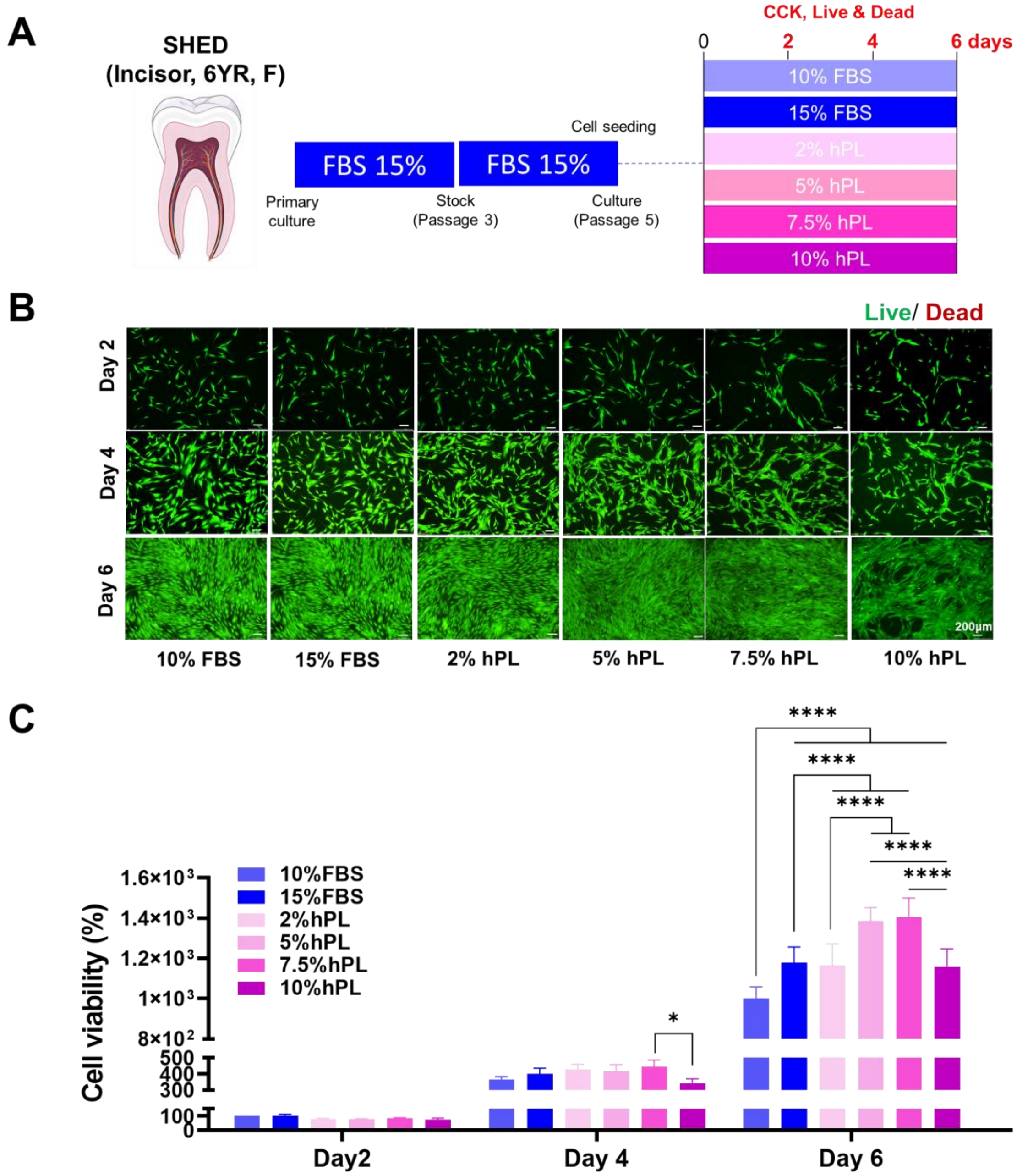

Figure S1. Cell proliferation effect on SHED according to the concentration of hPL. A) Schematic diagram illustrating the experimental protocol. SHED cultured under 10% FBS-contained mediumwas seeded in 10/15% FBS or 2/5/7.5/10% hPL supplemented medium. CCK and Live and dead assay were performed after 2, 4, and 6 days. B) Representative images of Live & Dead assay and C) CCK results at days 2, 4, and 6. There was no difference in proliferation rate among groups at day 2, 4. However, a tendency for increased proliferation was observed at day 6 depending on the concentration of FBS or hPL. Particularly, the 2% hPL group exhibited a similar result to the 15% FBS group, and the hPL group demonstrated the highest level in the 5% and 7.5% hPL groups, with a subsequent decrease in the 10% group.

Table S1. Primer sequences used in the qRT-PCR

Name	Forward	Reverse
COL1A	5′-CGTGACCAAAAACCAAAAGTGC-3′	5′-GGGTGGAGAAAGGAACAGAAA-3′
ALP	5′-ACACCTTGACTGTGGTTACT-3’	5’-CCTTGTAGCCAGGCCCGTTA-3′
DMP1	5′-GGACGGCTCTGAGTTCGA-3′	5′-TGGGTTTCCCTGCTGTTG-3′
DSPP	5′-AGAAGGACCTGGCCAAAAAT-3′	5’-TCTCCTCGGCTACTGCTGTT-3′
OCN	5′-AGACTCCGGCGCTACCTCAACAAT-3′	5′-CAGCTGTGCCGTCCATACT-3′
RUNX2	5′-GACCGACACAGCCATATAAA-3′	5'-TCCCTAACCTGAAACCAAAG-3′
PPAR-γ2	5′- AGCCTGCGAAAGCCTTTTGGTG-3′	5’-GGCTTCACATTCAGCAAACCTGG -3′
FABP4	5′- ACGAGAGGATGATAAACTGGTGG-3′	5′-GCGAACTTCAGTCCAGGTCAAC -3′
LPL	5′-CTGCTGGCATTGCAGGAAGTCT-3′	5′-CATCAGGAGAAAGACGACTCGG-3′
Col-IIA	5′-GTGACAAAGGAGAGGCTGGA-3′	5′-ACCTCTAGGGCCAGAAGGAC-3′
SOX9	5′-ATCTGAAGAAGACTGCGAG-3′	5′-TCAGAAGTCTCCAGAGCTTG-3′
ACAN	5′-CTGCTTCCGAGGCATTTCAG-3′	5′-CTTGGGTCACGATCCACTCC-3′
MASH-1	5′-CCAGTTGTACTTCAGCACC-3′	5′-TGCCACTTTGAGTTTGGAC-3′
NeuroD	5′-CCACGGATCAATCTTCTCAG-3′	5′-CATGATGTGAATGGCTATCG-3′
Nestin	5′-GCCCTGACCACTCCAGTTA-3′	5′-GGAGTCCTGGATTTCCTTCC-3′
GFAP	5′-GAGCACAAGGATGTGATGT-3′	5′-AGAGGCGGAGCAACTAT-3′
β-tubulin	5′-GAAGCCAGCAGTGTCTAAA-3′	5′-GAGGACGAGGCCATAAATAC-3′
GAPDH	5′-GAGCATCTCCCTCACAATTT-3′	5′-GGGTGCAGCGAACTTTAT-3′
